# Tumour Jagged1 expression as a prognostic marker of bevacizumab response and modulation of 5-fluorouracil efficacy through γ-secretase inhibition in colorectal cancer

**DOI:** 10.1093/gastro/goag012

**Published:** 2026-03-10

**Authors:** Olga María García-Valdeavero, Encarnación González-Flores, Raúl Ortiz, Julia Jiménez-López, Cristina Jiménez-Luna, Octavio Caba, Jose Prados, Consolación Melguizo

**Affiliations:** Biopathology and Regenerative Medicine Institute (IBIMER), Biomedical Research Center (CIBM), University of Granada, Av. del Conocimiento 19, 18016Granada, Spain; Instituto de Investigación Biosanitaria ibs.GRANADA, Av. de Madrid 15, 18012Granada, Spain; Medical Oncology Service, Virgen de las Nieves Hospital, Av. de las Fuerzas Armadas 2, 18014Granada, Spain; Biopathology and Regenerative Medicine Institute (IBIMER), Biomedical Research Center (CIBM), University of Granada, Av. del Conocimiento 19, 18016Granada, Spain; Instituto de Investigación Biosanitaria ibs.GRANADA, Av. de Madrid 15, 18012Granada, Spain; Faculty of Medicine, Department of Human Anatomy and Embryology, University of Granada, Av. de la Investigación 11, 18006Granada, Spain; Instituto de Bioquímica Vegetal y Fotosíntesis, University of Sevilla and CSIC, Av. Américo Vespucio 49, 41092 Sevilla, Spain; Biopathology and Regenerative Medicine Institute (IBIMER), Biomedical Research Center (CIBM), University of Granada, Av. del Conocimiento 19, 18016Granada, Spain; Instituto de Investigación Biosanitaria ibs.GRANADA, Av. de Madrid 15, 18012Granada, Spain; Faculty of Medicine, Department of Human Anatomy and Embryology, University of Granada, Av. de la Investigación 11, 18006Granada, Spain; Biopathology and Regenerative Medicine Institute (IBIMER), Biomedical Research Center (CIBM), University of Granada, Av. del Conocimiento 19, 18016Granada, Spain; Instituto de Investigación Biosanitaria ibs.GRANADA, Av. de Madrid 15, 18012Granada, Spain; Faculty of Medicine, Department of Human Anatomy and Embryology, University of Granada, Av. de la Investigación 11, 18006Granada, Spain; Biopathology and Regenerative Medicine Institute (IBIMER), Biomedical Research Center (CIBM), University of Granada, Av. del Conocimiento 19, 18016Granada, Spain; Instituto de Investigación Biosanitaria ibs.GRANADA, Av. de Madrid 15, 18012Granada, Spain; Faculty of Medicine, Department of Human Anatomy and Embryology, University of Granada, Av. de la Investigación 11, 18006Granada, Spain; Biopathology and Regenerative Medicine Institute (IBIMER), Biomedical Research Center (CIBM), University of Granada, Av. del Conocimiento 19, 18016Granada, Spain; Instituto de Investigación Biosanitaria ibs.GRANADA, Av. de Madrid 15, 18012Granada, Spain; Faculty of Medicine, Department of Human Anatomy and Embryology, University of Granada, Av. de la Investigación 11, 18006Granada, Spain

**Keywords:** colorectal cancer, Notch signalling, bevacizumab, JAG1, soluble ligand, targeted therapies

## Abstract

**Background:**

5-fluorouracil (5-FU)-based chemotherapy remains the backbone of metastatic colorectal cancer (CRC) treatment, although therapeutic resistance limits long-term benefit. Combination with bevacizumab improves outcomes in some patients, but biomarkers capable of predicting benefit are lacking. Notch signalling and altered expression of its ligand Jagged1 (JAG1) have been implicated in CRC progression, yet their relevance in bevacizumab-treated patients and their regulation by 5-FU remain unclear.

**Methods:**

JAG1 protein levels were quantified in tumour samples from patients with metastatic CRC (*n *= 60) by using enzyme-linked immunosorbent assay and correlated with clinical outcomes. *In vitro* experiments using HCT15 and SW480 CRC cell lines were used to assess the effects of combining 5-FU and the γ-secretase inhibitor *N*-[*N*-(3,5-difluorophenacetyl-l-alanyl)]-*S*-phenylglycine *t*-butyl ester (DAPT) on proliferation using 3-(4,5-dimethylthiazol-2-yl)-2,5-diphenyltetrazolium bromide assay. Notch pathway, stemness, epithelial–mesenchymal transition (EMT), and apoptosis markers were assessed by using quantitative PCR and/or Western blotting. The angiogenic capacity of the secretome was examined by using tube-formation assays.

**Results:**

Among patients receiving bevacizumab, those with low tumour JAG1 expression exhibited longer progression-free survival and time to progression than patients with high JAG1 expression. *In vitro*, DAPT plus 5-FU synergistically reduced CRC-cell viability, enhanced apoptosis and autophagy, reduced the expression of stemness and EMT-related genes, and impaired tube formation. Soluble JAG1 was detected in conditioned media, with higher levels following combination treatment in HCT15 cells.

**Conclusions:**

High tumour JAG1 expression identifies metastatic CRC patients with poorer outcomes when treated with a bevacizumab-containing regimen, supporting its potential as a prognostic biomarker. Mechanistically, Notch inhibition enhances the antitumour effects of 5-FU, suggesting that its combination with γ-secretase inhibitors may improve therapeutic efficacy in CRC.

## Introduction

Colorectal cancer (CRC) is the third-most common cancer in the world and the second leading cause of cancer death [1]. Unfortunately, the number of new cases of CRC and deaths from this malignancy are expected to increase by 2040 [2]. Despite advancements in early diagnostics and campaigns to promote regular screening in the general population, ≤30% of cases are diagnosed with metastatic disease and 20%–50% of patients with localized CRC will eventually develop metastases [3]. Meanwhile, the 5-year survival rate for patients with metastatic CRC is ∼13% [4].

5-Fluorouracil (5-FU) is a fundamental drug for the treatment of metastatic CRC. First-line 5-FU-based chemotherapies such as FOLFOX (5-FU, leucovorin, and oxaliplatin) or FOLFIRI (5-FU, leucovorin, and irinotecan) have improved survival outcomes and reduced distant recurrences in some patients [5, 6]. However, these therapies present important limitations, such as systemic toxicity and resistance development. Due to these drawbacks, the response rate to 5-FU remains low, at ∼15% of advanced CRC cases [7, 8].

The use of these treatments in combination with targeted therapies against vascular endothelial growth factor (VEGF) has also helped to significantly increase the survival in patients with metastatic CRC [9, 10]. Although side effects caused by anti-VEGF antibodies are normally well tolerated by patients, gastrointestinal perforation, hypertension, blood alterations, and skin disorders represent potential risks [11]. Additionally, issues such as intrinsic or acquired resistance are also observed when anti-VEGF therapies are administered together with 5-FU in patients with CRC [12]. Therefore, combinatorial treatments that improve the efficacy of 5-FU are urgently needed to increase survival of these patients.

The Notch signalling pathway is a highly relevant route implicated in numerous cellular processes, such as cell proliferation, apoptosis, and angiogenesis, among others [13, 14]. This pathway has been reported to be altered in several types of cancer, including ovarian [15], breast [16], pancreatic [17], and colorectal cancers [18]. The Notch pathway is activated when NOTCH1–4 receptors on the cell surface interact with their ligands, Delta-like ligands (DLL1, DLL3, and DLL4) and Jagged ligands (JAG1 and JAG2). After ligand binding, the Notch receptor undergoes two proteolytic cleavages: first by a disintegrin and metalloprotease domain 10 (ADAM10), which removes the extracellular domain of the receptor, and then by the γ-secretase complex, which releases the Notch intracellular domain (NICD). Eventually, NICD translocates into the nucleus and binds transcription factors such as Hes family bHLH transcription factor 1 (HES1), thereby modulating downstream pathways [19].

Some studies have demonstrated the role of *HES1* in tumour progression and metastasis in CRC [20, 21]. Strategies that inhibit Notch signalling and the expression of its downstream genes may contribute to improving clinical outcomes in this pathology. In this line, γ-secretase inhibitors (GSIs) impede the γ-secretase-mediated cleavage and the nuclear translocation of NICD, impairing the Notch pathway. Some GSIs have been shown to reduce cell growth and tumour progression in different types of cancer [22] and have been proposed for combinatorial treatments together with chemotherapy in order to reduce chemoresistance [23, 24]. Moreover, clinical trials using GSIs have shown positive results in different types of cancers [25]; however, more research is needed to successfully bring GSIs into the CRC clinical setting.

Here, we investigated the association between tumour JAG1 protein levels (one of the main Notch-activating ligands) and different clinical characteristics in patients with metastatic CRC. Furthermore, we analysed the potential benefit of combining the GSI *N*-[*N*-(3,5-Difluorophenacetyl-l-alanyl)]-*S*-phenylglycine t-butyl ester (DAPT) with 5-FU in *in vitro* CRC models.

## Patients and methods

### Clinical characteristics of patients

A total of 60 patients diagnosed with metastatic CRC at Virgen de las Nieves Hospital in Granada (Spain) were retrospectively selected and included in the study. These patients were identified and recruited between 2014 and 2017. Pathological staging was performed according to the TNM classification system. Formalin-fixed and paraffin-embedded (FFPE) tumour tissue samples were collected and processed for total protein extraction from all patients. The inclusion criteria were as follows: (i) patients >18 years of age, (ii) presence of at least one radiologically visible metastasis at the time of tissue sampling, (iii) tumour tissue samples obtained prior to any chemotherapy regimen, (iv) availability of sufficient FFPE tumour tissue to perform protein extraction, and (v) signed informed consent. This study adhered to the principles of the Declaration of Helsinki. The protocol was approved by the Biomedical Research Ethics Committee of the Andalusian Public Health System in Granada (protocol code PI19/01478; no. 2020522131049; 29 July 2020).

The mean age of the patients was 60.43 ± 11.30 years. Of the 60 patients, 25 (41.7%) were female and 29 (48.3%) were male. *KRAS* was mutated in 24 patients (40%), whereas 28 patients (46.7%) had wild-type *KRAS*. Moreover, 27 patients (45%) presented metastases in a single organ, while 26 (43.3%) had metastases in more than one organ. Regarding tumour localization, 14 patients (23.3%) had tumours in the rectum, 14 (23.3%) in the right colon, and 26 (43.3%) in the transverse colon. With respect to systemic therapy, 16 patients (26.7%) had received chemotherapy combined with bevacizumab, whereas 38 (63.3%) had received regimens that did not include an anti-angiogenic agent. The clinical characteristics of the cohort are summarized in Table 1.

**Table 1:** Characteristics of patients with metastatic CRC included in the study.

Characteristic	Number of patients (%)
**Age (mean ± SD)**	60.43 ± 11.30
**Sex**	
** Male**	29 (48.3)
** Female**	25 (41.7)
** Not available**	6 (10)
***KRAS* gene status**	
** Mutated**	24 (40)
** Wild-type**	28 (46.7)
** Not determined**	8 (13.3)
**Type of metastasis**	
** Single organ**	27 (45)
** Multiple organs**	26 (43.3)
** Not determined**	7 (11.7)
**Tumour location**	
** Rectum**	14 (23.3)
** Right colon**	14 (23.3)
** Transverse colon**	26 (43.3)
** Not determined**	6 (10)
**Chemotherapy treatment**	
** Without anti-angiogenic therapy**	38 (63.3)
** With anti-angiogenic therapy**	16 (26.7)
** Not determined**	6 (10)

### Analysis of JAG1 levels in human CRC tissue

For protein extraction from FFPE tumour tissue, samples were deparaffinized by immersion in xylene. Once the paraffin was removed, the tumour tissue was rehydrated by using a graded series of ethanol solutions (100%, 95%, and 70%) and finally immersed in water. To determine the JAG1 protein levels in the patient-derived tumour tissue, whole lysate samples were analysed by using the Human Jagged1 ELISA Kit (Sigma-Aldrich, St Louis, MO, USA). JAG1 tumour levels were expressed as nanograms of JAG1 per milligram of total protein. To examine the relationship between JAG1 expression and clinical parameters, patients were categorized into two groups based on the 50th percentile (median). Those with JAG1 levels below this threshold were classified as having low expression, whereas those with values above the threshold were classified as having high expression.

### Cell culture and reagents

Two established human CRC cell lines, HCT15 and SW480, were purchased from the American Type Culture Collection (Manassas, VA, USA). Cells were cultured in Dulbecco’s Modified Eagles Medium (Sigma-Aldrich) supplemented with 10% heat-inactivated fetal bovine serum (FBS; Thermo Fisher Scientific, Waltham, MA, USA) and 1% penicillin/streptomycin (Sigma-Aldrich) at 37°C in a humidified atmosphere containing 5% CO_2_.

Human umbilical vein endothelial cells (HUVECs), used in the tube-formation assay, were obtained from the Centre for Scientific Instrumentation (CIC—University of Granada, Spain). HUVECs were cultured in 0.1% gelatine-coated flasks by using Endothelial Cell Growth Medium-2 (EGM^TM^-2; Lonza, Basel, Switzerland), supplemented with EGM^TM^-2 SingleQuots (Lonza), which included FBS, human epidermal growth factor, VEGF, R3-insulin-like growth factor-1, ascorbic acid, hydrocortisone, human fibroblast growth factor-β, heparin, and gentamicin/amphotericin-B, following the manufacturer’s instructions.

CRC cell lines were treated with 5-FU (Sigma-Aldrich) and/or the GSI DAPT (Santa Cruz Biotechnology, Dallas, TX, USA), as indicated in each experiment. An aqueous solution of 5-FU was prepared in distilled water and stored at 4°C. A 100 mM DAPT stock solution was prepared in dimethyl sulfoxide (DMSO; Sigma-Aldrich) and stored at −20°C.

### Cell viability analysis

HCT15 and SW480 cells previously cultured in monolayer were harvested by using trypsinization (0.25% trypsin–EDTA solution; Sigma-Aldrich) and seeded in 96-well plates (Thermo Fisher Scientific) at a density of 2 × 10^3^ cells/well. First, the cells were exposed to increasing concentrations of 5-FU (0.1–20 μM) for 48 and 72 h to estimate the inhibitory concentrations (ICs) of the 5-FU causing approximately 5%, 10%, and 25% growth inhibition (IC_5_, IC_10_, and IC_25_, respectively). These ICs were subsequently used in proliferation assays in the presence or absence of DAPT (25 μM). An equivalent concentration of DMSO (0.1% v/v) was used as a vehicle control.

To study the impact of DAPT on 5-FU efficacy, two experimental approaches were employed. In the first (simultaneous treatment), CRC cells were treated 24 h after seeding with 5-FU and DAPT added simultaneously or with either agent alone and incubated for 72 h. In the second approach (sequential or presensitizing treatment), cells were first treated with DAPT alone for 24 h to presensitize them, after which 5-FU was added and treatment continued for an additional 48 h. In both experimental settings, cell proliferation was assessed 96 h after seeding by using the 3-(4,5-dimethylthiazol-2-yl)-2,5-diphenyltetrazolium bromide (MTT) assay (Sigma-Aldrich), following the manufacturer’s instructions. Fresh medium containing 10% MTT solution was added to each well and the plates were incubated for 4 h at 37°C in a humidified atmosphere containing 5% CO_2_. After incubation, the medium was carefully replaced with 100 μL of a DMSO/Sørensen’s phosphate buffer mixture (9:1 v/v; pH 10.5) to dissolve the formazan crystals. Absorbance was measured at 570 and 630 nm by using the 800™ TS Absorbance Reader (BioTek, Santa Clara, CA, USA) with Gen5 software (BioTek). Each condition was tested in five technical replicates and a minimum of three independent biological replicates were performed.

Cell proliferation was calculated as follows: % proliferation = [absorbance of sample (570–630)/absorbance of control (570–630)] ×100.

### Real-time quantitative PCR

Total RNA from the HCT15 and SW480 cells was isolated by using TRIzol reagent (Sigma-Aldrich) followed by purification via the RNeasy Mini Kit (Qiagen, Hilden, Germany). RNA concentration and purity were assessed by using a NanoDrop 2000 spectrophotometer (Thermo Fisher Scientific). Two micrograms of RNA were reverse-transcribed into complementary DNA by using the SuperScript™ III First-Strand Synthesis SuperMix (Thermo Fisher Scientific).

RT–qPCR was performed via a StepOnePlus Real-Time PCR System (Applied Biosystems, Waltham, MA, USA) using TB Green Premix Ex Taq II (Tli RNase H Plus, Takara, Kusatsu, Shiga, Japan). The expression of *JAG1*, *HES1*, Snail family transcriptional repressor 1 (*SNAIL*), Octamer-binding protein 4 (*OCT-4*), and Nanog Homeobox (*NANOG*) was analysed. Gene expression was normalized to Hypoxanthine phosphoribosyltransferase 1 (*HPRT1*), which was selected as the most stable reference gene in our experimental setting, as determined by using the RefFinder tool [26] (Supplementary Fig. S1A and B).

Primers were designed and synthesized by Sigma-Aldrich. The primer sequences are listed in Table 2 and Supplementary Table S1. Relative gene expression was calculated by using the 2^−ΔΔCt^ method. Each condition was analysed in technical triplicates and all experiments were repeated at least twice as independent biological replicates.

**Table 2:** Primers used in the RT–qPCR experiments.

Gene	Primer sequence	Tm (°C)
*HPRT1*	F- TGACACTGGCAAAACAATGCA	67.5
	R- GGTCCTTTTCACCAGCAAGCT	66.6
*JAG1*	F- ACTACTACTATGGCTTTGGC	55.1
	R- ATAGCTCTGTTACATTCGGG	58.3
*HES1*	F- GCCTATTATGGAGAAAAGACG	59.3
	R- CTATCTTTCTTCAGAGCATCC	57.2
*SNAIL*	F- AACAATGTCTGAAAAGGGAC	58.1
	R- ATAGTTCTGGGAGACACATC	55.4
*OCT-4*	F- GATCACCCTGGGATATACAC	58.1
	R- GCTTTGCATATCTCCTGAAG	59.1
*NANOG*	F- CCAGAACCAGAGAATGAAA	60.1
	R- TGGTGGTAGGAAGAGTAAA	55.9

F- = forward, R- = reverse, Tm = melting temperature.

### Conditioned medium generation

HCT15 and SW480 cell lines were seeded in 24-well plates at a density of 1 × 10^5^ cells/well and treated 24 h after seeding with 5-FU at its IC_10_ (established for 72 h of exposure), DAPT at 25 μM, or a combination of both. These doses were non-cytotoxic under the short-term conditions used for conditioned media (CM) generation (24 h). Untreated and DMSO controls were also included in the corresponding experiments. After 24 h of treatment, the culture medium was replaced by fresh medium supplemented with 0.5% FBS and maintained for an additional 24 h. Finally, CM was collected, centrifuged to remove cell debris, and stored at −80°C until use.

### Tube-formation assay

HUVECs were grown in EGM-2 medium supplemented as described above. Then, 12 h before the start of the tube-formation experiments, the culture medium was refreshed without FBS and VEGF supplements. Subsequently, HUVECs were seeded in 96-well plates previously coated with Matrigel (Corning, NY, USA) at a cell density of 5 × 10^4^ cells/well and cultured with CM for 12 h. Cells were incubated with calcein (Santa Cruz Biotechnology) at 2 μM for 30 min and tube formation was visualized and photographed under fluorescent microscopy by using the DM IL LED microscope (Leica Microsystems, Wetzlar, Germany). The vessel percentage area, vessel length, and average lacunarity parameters were analysed by using the image-processing software AngioTool v0.6a (National Cancer Institute, USA).

### Western blot studies

HCT15 and SW480 cell lines were seeded in six-well plates at densities of 7 × 10^4^ and 9 × 10^4^ cells/well, respectively. With post-seeding for 24 h, cells were treated with 5-FU (IC_10_), either alone or in combination with DAPT (25 μM). After 72 h of treatment, total protein was extracted by using RIPA lysis buffer (G-Biosciences, Saint Louis, MO, USA) containing a protease inhibitor cocktail (Thermo Fisher Scientific). Protein concentrations were determined by using the Bradford assay (PanReact AppliChem, Castellar del Vallès, Barcelona, Spain) and 40 μg of pre-denatured protein (heated at 95°C for 5 min) was used for Western blot analysis.

To study soluble JAG1 (sJAG1) and vascular endothelial growth factor A (VEGFA), CM derived from HCT15 and SW480 cells treated as previously described was incubated with 5 µL/mL StrataClean Resin (Agilent Technologies, Santa Clara, CA, USA) overnight at 4°C under gentle agitation. The resin was then recovered by using centrifugation at 4,000 rpm for 4 min. Bound proteins were eluted by using 20 μL of Laemmli buffer and heated at 95°C for 5 min. Finally, the samples were centrifuged at 4,000 rpm for 4 min and the resulting supernatants were used for Western blot analysis.

Electrophoresis was performed on a 12% acrylamide SDS–PAGE gel by using the Mini-PROTEAN Tetra Vertical Electrophoresis Cell (Bio-Rad, Hercules, CA, USA). Following separation, proteins were transferred to a nitrocellulose membrane by using the Trans-Blot Turbo RTA Midi 0.2 µm Nitrocellulose Transfer Kit and the Trans-Blot Turbo Transfer System (Bio-Rad). Membranes were blocked for 1–2 h at room temperature by using 5% fat-free dry milk dissolved in 1× Tris buffered saline (TBS) containing 0.1% Tween 20 (TBS-T; Bio-Rad).

Primary antibody incubation was carried out overnight at 4°C with the following antibodies: anti-Jagged1 (sc-390177; Santa Cruz Biotechnology; 1:250), anti-HES1 (HY-P80155; MedChemExpress, Monmouth Junction, NJ, USA; 1:1,000), anti-PARP1 (ab32138; Abcam, Cambridge, MA, USA; 1:1,000), anti-LC3β (NB100-2220; Novus Biologicals, Centennial, CO, USA; 1:1,000), anti-Jagged1 N-terminal (AF1277; R&D Systems, Minneapolis, MN, USA; 1 μg/mL), and anti-VEGFA (ab46154; Abcam; 1:1,000). The next day, membranes were incubated for 1 h at room temperature with the following peroxidase-conjugated secondary antibodies: anti-mouse IgGκ BP-HRP (sc-516102; Santa Cruz Biotechnology; 1:5,000), anti-rabbit IgG-HRP (sc-2357; Santa Cruz Biotechnology; 1:5,000), and anti-goat IgG-HRP (sc-2020; Santa Cruz Biotechnology; 1:5,000).

For the loading control, β-actin expression was detected by using a peroxidase-conjugated anti-β-actin antibody (A3854; Sigma-Aldrich; 1:25,000). For CM protein normalization, a consistent Ponceau S-stained band was used as the reference. Finally, bands of interest were visualized by using Amersham ECL Prime (Thermo Fisher Scientific) on a ChemiDoc MP Imaging System (Bio-Rad) and analysed by using Image Lab Software (Bio-Rad).

### Statistical analysis

The Kaplan–Meier method was used to estimate and compare the time to progression (TTP) and progression-free survival (PFS) of patients. The log-rank (Mantel–Cox) or Gehan–Breslow–Wilcoxon tests were used to analyse differences between survival curves, as appropriate. Hazard ratios (HRs) with 95% confidence intervals (CIs) were calculated by using the Mantel–Haenszel or log-rank tests. In some analyses, effect sizes were calculated by using Cohen’s *d*, interpreted as follows: *d *< 0.20, no effect; *d *= 0.20–0.49, small effect; *d *= 0.50–0.79, medium effect; and *d *≥ 0.80, large effect.

For *in vitro* experiments, two-tailed unpaired Student’s *t*-tests were used for comparisons between two groups, whereas one-way ANOVA was used for comparisons between three or more groups. A one-sample *t*-test was used to assess whether the normalized values significantly differed from 1. All statistical analyses were conducted by using GraphPad Prism 9.3 software. A *P* value of <0.05 was considered statistically significant. All results were expressed as mean ± SD.

The synergistic effect of the combination of DAPT and 5-FU was analysed by using SynergyFinder Plus software. The high single-agent (HSA) reference model was used to calculate the synergy scores, following the software recommendations. This model assumes that the expected effect of a drug combination equals the maximal effect produced by either drug alone at the corresponding concentrations; synergy is inferred when the observed effect exceeds this reference value. According to this analysis tool, synergy scores of >10 indicate a probable synergistic interaction between the two drugs, values between −10 and 10 indicate an additive interaction, and scores below −10 reflect an antagonistic effect [27].

## Results

### Anti-angiogenic therapy increases survival in patients with metastatic CRC

**Figure 1:**
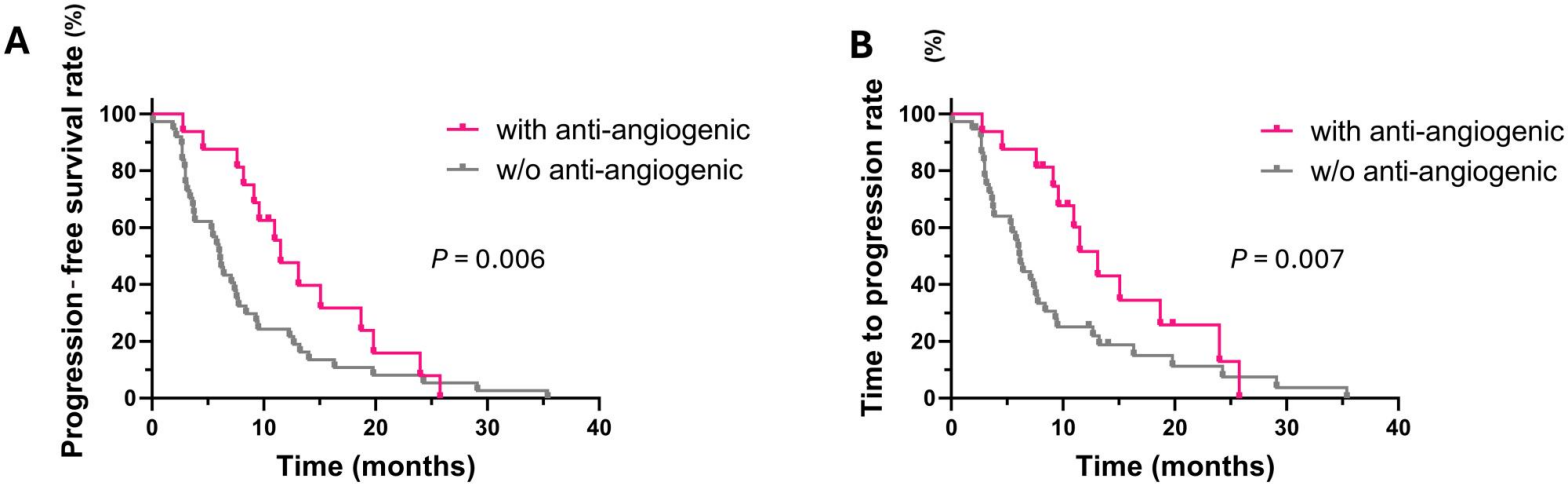
Kaplan–Meier plots of **(**A) PFS and (B) TTP in patients with metastatic CRC treated with chemotherapy with or without bevacizumab. Differences between curves were analysed by using the Gehan–Breslow–Wilcoxon test. *P *< 0.05 was considered statistically significant. PFS, progression-free survival; TTP, time to progression; CRC, colorectal cancer; w/o, without.

Firstly, we evaluated the impact of combining bevacizumab with chemotherapy in our patient cohort. As we expected, patients receiving this combination (*n *= 16) showed better survival outcomes than those not receiving anti-angiogenic therapy (*n *= 37; one patient was excluded due to missing PFS/TTP data). Specifically, patients treated with bevacizumab exhibited significantly longer PFS (median 11.5 vs 6.1 months; *P *= 0.006) and TTP (median 13.1 vs 6.1 months; *P *= 0.007) than those who did not (Fig. 1A and B). The HRs for PFS and TTP were 0.56 (95% CI: 0.32–0.99) and 0.53 (95% CI: 0.29–0.97), respectively, indicating a lower risk of disease progression in patients with metastatic CRC treated with this combinatorial regimen. These results demonstrate that adding the anti-angiogenic drug bevacizumab to chemotherapy significantly provides survival benefits in patients with metastatic CRC.

### JAG1 levels are associated with prognosis in patients treated with bevacizumab

**Figure 2:**
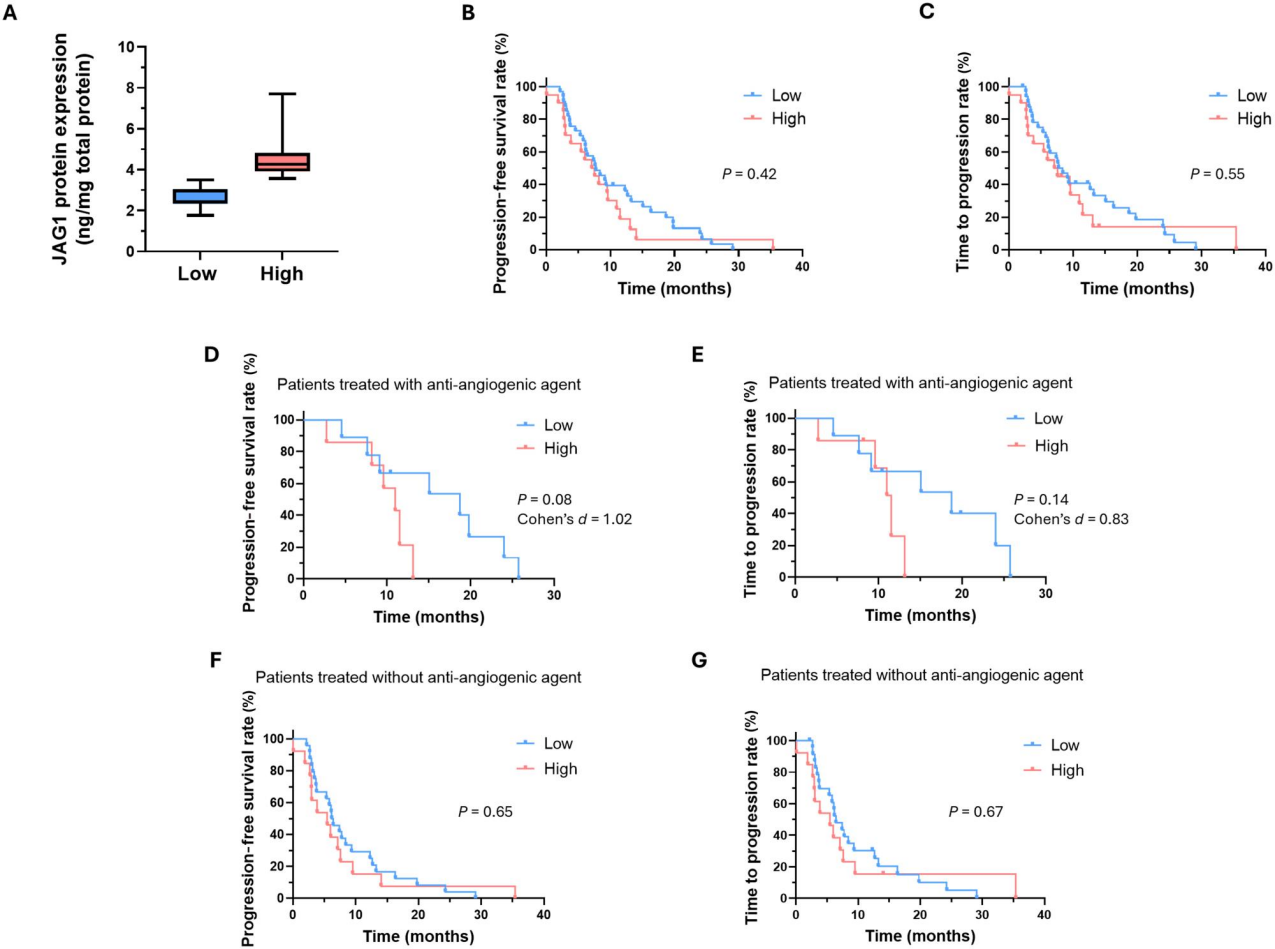
Analysis of prognosis-related parameters in patients with low vs high JAG1 expression. (A) Classification of metastatic CRC patients according to low and high JAG1 protein levels (cut-off value: 50th percentile). (B–G) Kaplan–Meier plots showing PFS and TTP in patients with low vs high JAG1 protein expression considering (B, C) the total cohort, (D, E) patients treated with bevacizumab, and (F, G) patients without bevacizumab in the therapeutic regimen. Cohen’s *d* analysis is shown. Differences between survival curves were analysed by using log-rank test. *P *< 0.05 was considered statistically significant. CRC, colorectal cancer; JAG1, Jagged1; PFS, progression-free survival; TTP, time to progression.

To examine the relationship between the tumour JAG1 levels and clinical variables, we first divided the 60 patients included in this study into two groups based on their protein expression levels (Fig. 2A). According to the established cut-off value (3.55 ng JAG1/mg total protein), we found that 22 patients (36.7%) exhibited high JAG1 expression, whereas 38 patients (63.3%) showed low expression.

We next analysed the influence of JAG1 on the prognosis for patients with metastatic CRC by examining the relationship between the JAG1 protein expression and several clinical parameters. When patients were stratified into low and high JAG1 expression groups, no significant differences in the median PFS (7.7 vs 7.3 months; *P *= 0.42) or TTP (8.0 vs 7.3 months; *P *= 0.55) were observed (Fig. 2B and C). However, among patients treated with chemotherapy plus bevacizumab, those with low JAG1 levels (*n *= 9) tended to have longer PFS (median 18.6 vs 10.9 months; *P *= 0.08) and TTP (median 18.6 vs 11.5 months; *P *= 0.14) compared with patients with high JAG1 (*n *= 7) (Fig. 2D and E). Although these differences did not reach statistical significance, the *P* values suggested a trend toward improved outcomes and the curves were clearly separated. We therefore calculated the effect sizes by using Cohen’s *d*, obtaining values of 1.02 for PFS and 0.83 for TTP, indicating large effects. This suggests that the absence of statistical significance was likely attributable to the small sample size in each group.

By contrast, in patients not receiving bevacizumab, PFS and TTP were similar between those with low (*n *= 24) and high (*n *= 13) JAG1 levels (median PFS: 6.2 vs 5.4 months, *P *= 0.65; median TTP: 6.4 vs 5.4 months, *P *= 0.67) (Fig. 2F and G), with negligible effect sizes (*d *= 0.15 and *d *= 0.23 for PFS and TTP, respectively). One patient was excluded from this analysis due to missing PFS/TTP data. Together, these data suggest that, while JAG1 levels do not appear to influence the prognosis for patients not receiving the anti-angiogenic agent, those treated with chemotherapy plus bevacizumab may derive greater benefit if they have low tumour JAG1 expression.

### DAPT combined with 5-FU enhances proliferation inhibition in CRC cell lines

First, we determined the IC_5_, IC_10_, and IC_25_ values of 5-FU for HCT15 and SW480 cells after 48 and 72 h of treatment. These values were subsequently used in proliferation assays with and without DAPT presensitization (Table 3).

**Table 3:** Determination of IC of 5-FU in HCT15 and SW480 cell lines under two different exposure schedules.

Treatment schedule	Cell line	IC_5_ (μM)	IC_10_ (μM)	IC_25_ (μM)
Simultaneous^a^	HCT15	0.20 ± 0.06	0.48 ± 0.34	3.47 ± 0.21
	SW480	1.14 ± 0.45	1.33 ± 0.54	3.21 ± 0.40
Sequential^b^	HCT15	0.19 ± 0.25	0.41 ± 0.35	4.17 ± 0.28
	SW480	1.01 ± 0.01	2.72 ± 1.96	9.23 ± 3.87

Values of ICs calculated from dose–response curves as the concentration of 5-FU that inhibits cell survival by 5% (IC_5_), 10% (IC_10_), and 25% (IC_25_) compared with the untreated control.

a 5-FU was added 24 h after seeding and maintained for 72 h, simulating the simultaneous-treatment schedule in which both 5-FU and DAPT are added at the same time.

b 5-FU was added 48 h after seeding and maintained for 48 h, simulating the sequential treatment (i.e. presensitizing treatment) in which DAPT is added 24 h after seeding and remains for 72 h, while 5-FU is added 24 h later (i.e. 48 h post-seeding) and remains for 48 h. In both cases, the total duration from cell seeding to viability assessment was 96 h. Data are expressed as means ± SD of three independent experiments.

**Figure 3:**
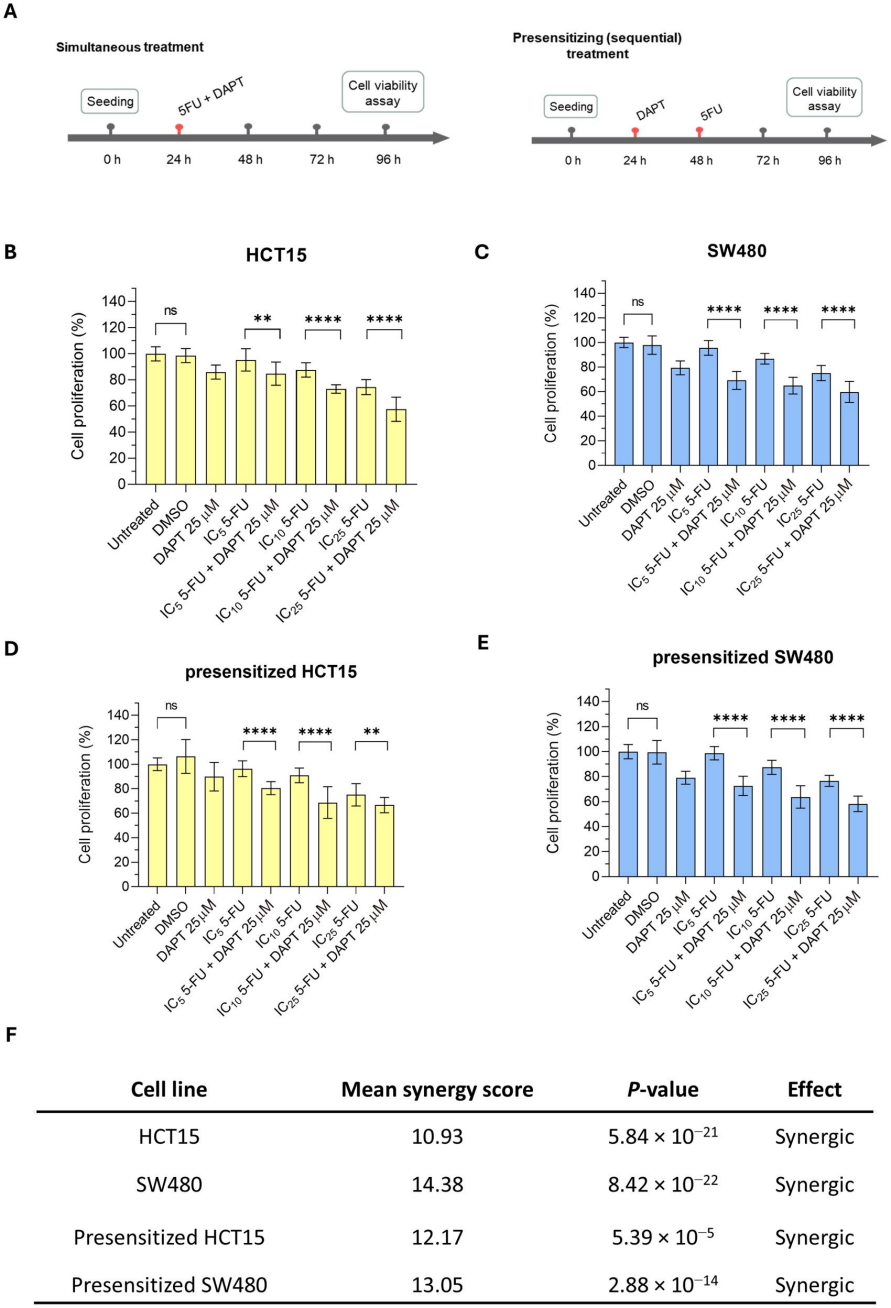
Effect of 5-FU in combination with DAPT on cell proliferation in CRC cell lines. (A) Schematic representation of simultaneous and presensitizing (sequential) treatment schedules used in the *in vitro* experiments. (B) HCT15 and (C) SW480 cells were treated 24 h after seeding with the indicated concentrations of 5-FU and DAPT, either individually or in combination, and incubated for 72 h (simultaneous treatment). (D) HCT15 and (E) SW480 cells were presensitized with DAPT for 24 h after seeding, followed by exposure to 5-FU at the indicated concentrations for an additional 48 h (sequential or presensitizing treatment). Untreated and DMSO-treated cells (0.1%) were included as controls. Cell viability was assessed by using the MTT assay. Differences between combination treatments and 5-FU alone were analysed by using a two-tailed unpaired Student’s *t*-test. Results are presented as mean ± SD of at least three independent experiments. (F) Synergy scores for drug combinations using 5-FU (at IC_5_, IC_10_, and IC_25_ doses) and DAPT (25 µM) in HCT15 and SW480 cells. Treatments followed the same simultaneous and presensitizing schemes as described in (A–E). Synergy was calculated by using the HSA model in the SynergyFinder Plus software. Statistical significance is indicated by *P < 0.05, **P < 0.01, ***P < 0.001, ****P < 0.0001. SD, standard deviation; 5-FU, 5-fluorouracil; CRC, colorectal cancer; DAPT, *N*-[*N*-(3,5-difluorophenacetyl-l-alanyl)]-*S*-phenylglycine *t*-butyl ester; DMSO, dimethyl sulfoxide; IC_5_, inhibitory concentration reducing viability by 5%; IC_10_, inhibitory concentration reducing viability by 10%; IC_25_, inhibitory concentration reducing viability by 25%; HSA, highest single-agent; ns, not significant; MTT, 3-(4,5-dimethylthiazol-2-yl)-2,5-diphenyltetrazolium bromide.

To investigate whether DAPT enhances the antitumour effect of 5-FU, HCT15 and SW480 cells were treated either simultaneously or sequentially (presensitization) with DAPT (25 μM) and 5-FU at IC_5_, IC_10_, and IC_25_ doses (Fig. 3A). Individual treatments with 5-FU, DAPT, DMSO, and untreated controls were also included. As shown in Fig. 3B and C, simultaneous treatment with 5-FU and DAPT reduced cell proliferation compared with treatment with 5-FU alone in both cell lines. In HCT15 cells, IC_10_ 5-FU reduced the proliferation to 88%, which further decreased to 73% upon the addition of DAPT (*P *< 0.0001; Fig. 3B). A similar pattern was observed at IC_25_, with a significant 17% reduction in proliferation under the combined treatment (*P *< 0.0001). Likewise, in SW480 cells, the antiproliferative effect of 5-FU was significantly enhanced at all doses when DAPT was added (Fig. 3C), with reductions of 26%, 22%, and 15% for IC_5_, IC_10_, and IC_25_ combinations, respectively (*P *< 0.0001).

Consistent results were obtained when the cells were presensitized with DAPT before the addition of 5-FU. In HCT15 cells, all doses of 5-FU induced greater proliferation inhibition when combined with DAPT (Fig. 3D), with the largest difference observed with DAPT plus IC_10_ 5-FU (22% reduction; *P *< 0.0001). In SW480 cells, the strongest effects were observed with DAPT plus IC_5_ and IC_10_ 5-FU, leading to significant reductions of 26% (*P *< 0.0001) and 24% (*P *< 0.0001), respectively (Fig. 3E). No significant differences were detected between untreated cells and those treated with DMSO as a vehicle control in any experiment.

We further examined whether the combination of DAPT and 5-FU exerted a synergistic effect by using SynergyFinder Plus. The HSA synergy scores for DAPT plus 5-FU exceeded 10 in both cell lines (Fig. 3F). Under simultaneous treatment, the mean synergy scores were 10.93 (*P *= 5.84 × 10^−21^) and 14.38 (*P *= 8.42 × 10^−22^) for HCT15 and SW480 cells, respectively. In presensitized cells, the HSA synergy scores were 12.17 (*P *= 5.39 × 10^−5^) and 13.05 (*P *= 2.88 × 10^−14^) for HCT15 and SW480, respectively. These findings indicate that the addition of DAPT to 5-FU potentiates the activity of 5-FU and synergistically reduces cell proliferation in the tested cell lines, both under simultaneous treatment and following presensitization.

### 5-FU upregulates Notch pathway genes in CRC cell lines

After confirming the synergistic effect of 5-FU and DAPT, we investigated whether 5-FU alone could influence Notch signalling activity. To address this, we assessed the relative expression of two key genes involved in this pathway, *HES1* and *JAG1*, in HCT15 and SW480 cells following exposure to increasing concentrations of 5-FU (IC_10_ and IC_25_ doses) for 72 h.

**Figure 4:**
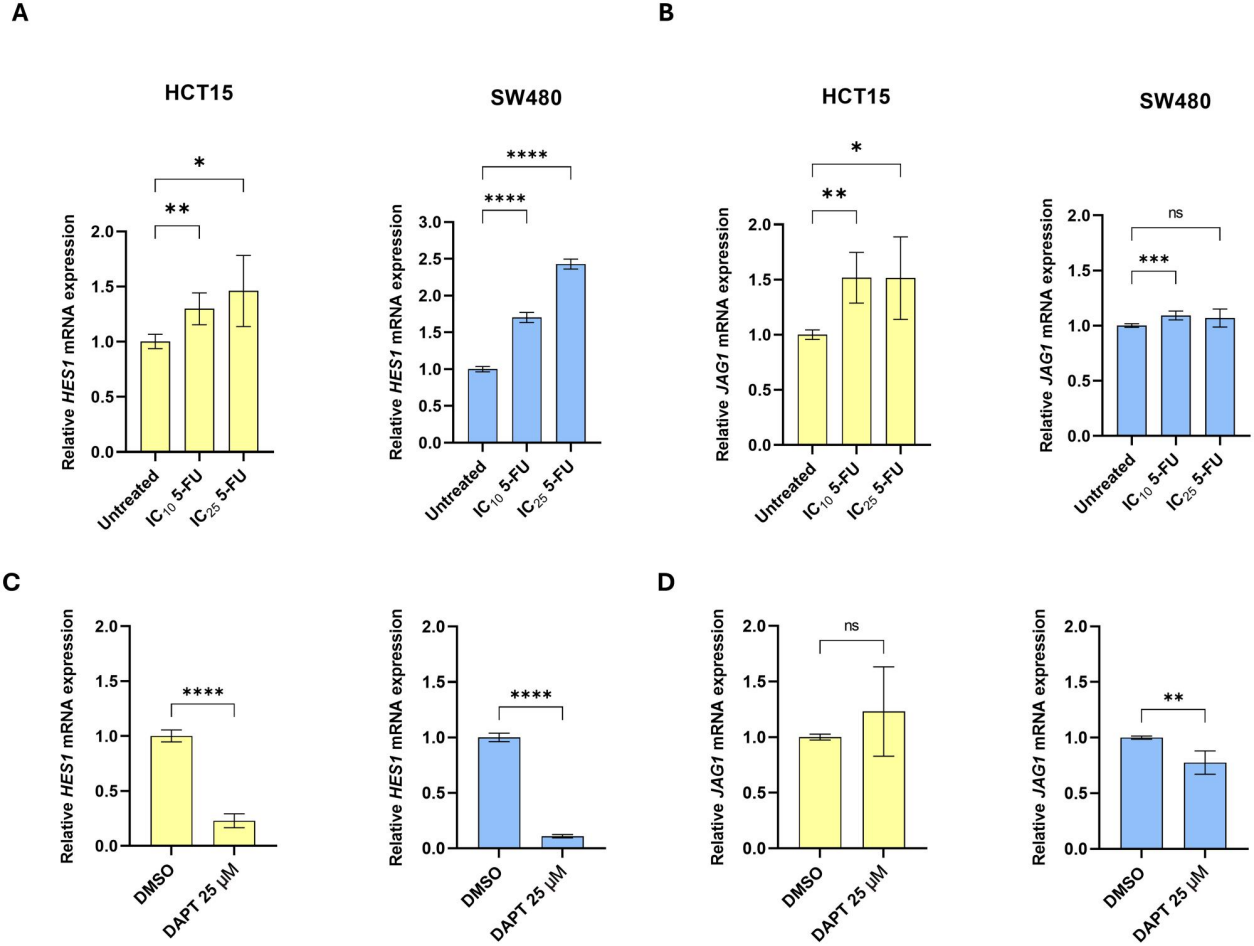
Impact of 5-FU and DAPT on the expression of Notch-related genes in CRC cell lines. Relative mRNA-expression levels of (A) *HES1* and (B) *JAG1* in HCT15 and SW480 cells following treatment with increasing concentrations of 5-FU for 72 h. Effect of DAPT (25 µM, 72 h) on the expression of (C) *HES1* and (D) *JAG1* compared with vehicle-treated controls (0.1% DMSO). Gene-expression levels were quantified by using RT–qPCR and normalized to the endogenous control *HPRT1*. Data were analysed by using (A, B) one-way ANOVA or (C, D) two-tailed unpaired Student’s *t*-test. Results are shown as mean ± SD of at least two independent experiments. Statistical significance is indicated by *P < 0.05, **P < 0.01, ***P < 0.001, ****P < 0.0001. ns, not significant; SD, standard deviation; 5-FU, 5-fluorouracil; CRC, colorectal cancer; DAPT, *N*-[*N*-(3,5-difluorophenacetyl-l-alanyl)]-*S*-phenylglycine *t*-butyl ester; DMSO, dimethyl sulfoxide; IC_10_, inhibitory concentration reducing viability by 10%; IC_25_, inhibitory concentration reducing viability by 25%; HES1, Hes family bHLH transcription factor 1; HPRT1, Hypoxanthine phosphoribosyltransferase 1; IC, inhibitory concentration; JAG1, Jagged1.

As shown in Fig. 4A, we observed a modest but significant increase in *HES1* expression in HCT15 cells treated with IC_10_ (1.3-fold, *P *= 0.0021) and IC_25_ (1.5-fold, *P *= 0.0193) doses of 5-FU compared with basal levels. In contrast, SW480 cells showed a clearer dose-dependent increase in *HES1* expression, which was significant at both IC_10_ (1.7-fold, *P *< 0.0001) and IC_25_ (2.4-fold, *P *< 0.0001).

Regarding *JAG1* expression (Fig. 4B), the two cell lines displayed distinct responses to 5-FU. In HCT15 cells, 5-FU induced a significant upregulation of *JAG1* at both IC_10_ (1.5-fold, *P *= 0.0021) and IC_25_ (1.5-fold, *P *= 0.0214). However, in SW480 cells, the *JAG1* expression remained largely unchanged, suggesting that the observed increase in *HES1* might have been mediated through alternative Notch-related mechanisms rather than the JAG1/Notch axis.

### DAPT treatment modulates Notch-related gene expression

Following the analysis of 5-FU effects on the expression of *HES1* and *JAG1*, we next examined how DAPT influences the expression of these Notch-related genes. As expected, DAPT treatment led to a strong downregulation of *HES1* in both HCT15 and SW480 cells, with approximately 5-fold and 10-fold decreases, respectively, compared with vehicle-treated controls (*P *< 0.0001; Fig. 4C).

Regarding *JAG1* expression (Fig. 4D), DAPT did not induce significant changes in HCT15 cells (*P *= 0.2188). However, in SW480 cells, the *JAG1* expression was significantly reduced by ∼1.4-fold compared with that in DMSO-treated controls (*P *= 0.0032).

### The combination of 5-FU and DAPT downregulates Notch pathway components

We investigated whether combining DAPT with 5-FU could counteract the 5-FU-induced upregulation of Notch signalling genes. For these experiments, CRC cells were simultaneously treated with DAPT (25 µM) and 5-FU at IC_10_ doses, as this was the lowest concentration at which we had previously observed significant changes in the expression of Notch-related genes and a reduction in CRC-cell proliferation.

**Figure 5:**
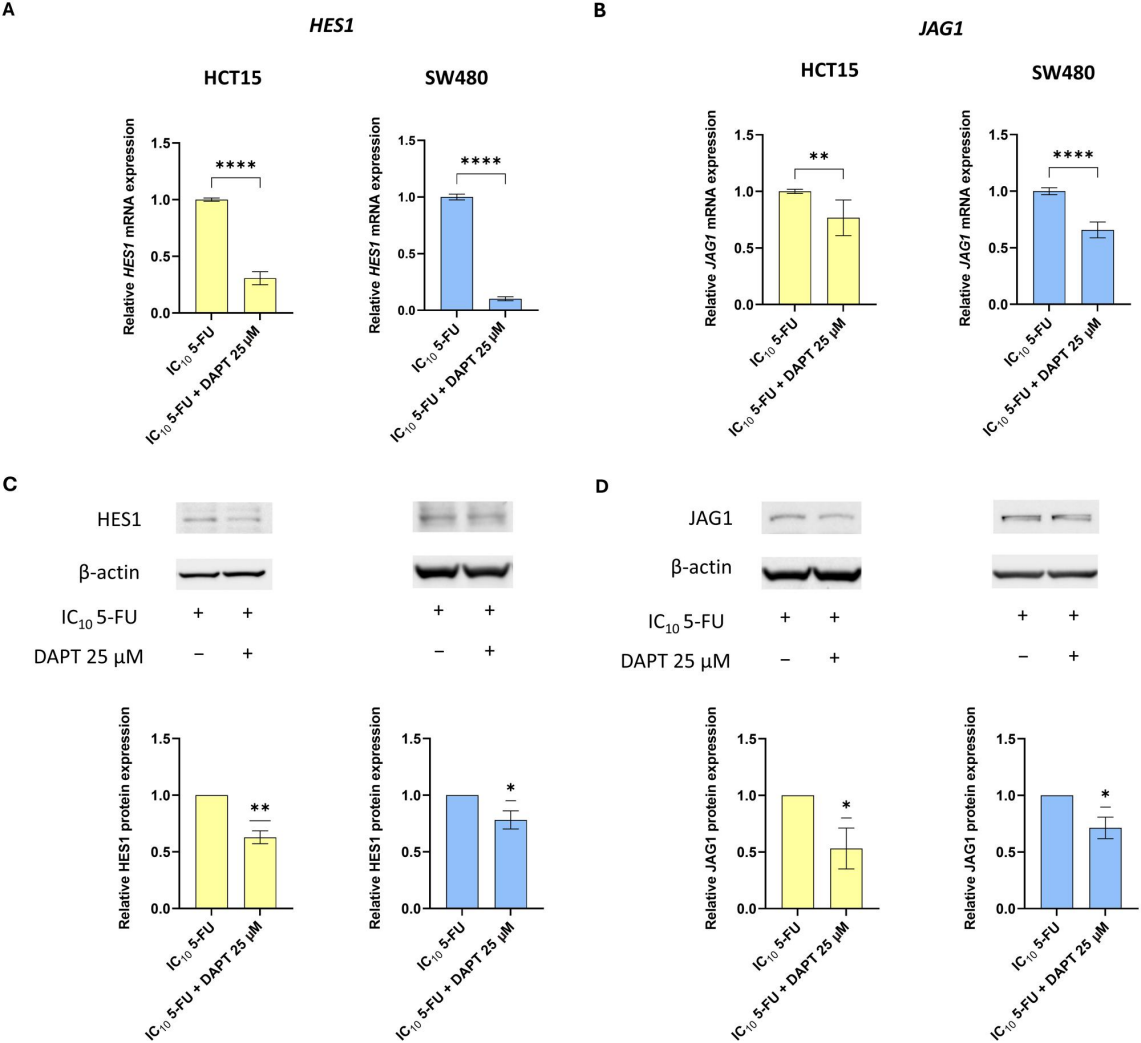
Modulation of Notch-related genes and proteins following treatment with 5-FU and DAPT in CRC cells. Relative mRNA levels of (A) *HES1* and (B) *JAG1* in HCT15 and SW480 cells treated with 5-FU alone or in combination with DAPT for 72 h. Treatments were performed using the simultaneous-treatment schedule. Gene expression was evaluated by using RT–qPCR and normalized to *HPRT1*. Differences between conditions were analysed by using a two-tailed unpaired Student’s *t*-test. Data are presented as mean ± SD of at least two independent experiments. Protein levels of (C) HES1 and (D) JAG1 were assessed in HCT15 and SW480 cells under the same treatment conditions as described in (A, B). Protein expression was evaluated by using Western blot; representative blots and densitometric quantification normalized to β-actin are shown. Differences between conditions were analysed by using a one-sample *t*-test. Data are presented as mean ± SD of three independent experiments. Statistical significance is indicated by *P < 0.05, **P < 0.01, ***P < 0.001, ****P < 0.0001. SD, standard deviation; 5FU, 5-fluorouracil; CRC, colorectal cancer; DAPT, *N*-[*N*-(3,5-difluorophenacetyl-l-alanyl)]-*S*-phenylglycine *t*-butyl ester; DMSO, dimethyl sulfoxide; IC_10_, inhibitory concentration reducing viability by 10%; HES1, Hes family bHLH transcription factor 1; HPRT1, Hypoxanthine phosphoribosyltransferase 1; JAG1, Jagged1.

Interestingly, the combined treatment led to a marked decrease in *HES1* expression compared with 5-FU alone (Fig. 5A), with 3.2-fold and 10-fold reductions in HCT15 and SW480 cells, respectively (*P *< 0.0001). Similarly, the *JAG1* expression was significantly reduced in both cell lines upon combination treatment (Fig. 5B), though to a lesser extent than the *HES1* expression (1.3-fold reduction in HCT15, *P *= 0.0021; 1.52-fold reduction in SW480, *P < *0.0001).

Consistently with the mRNA data, Western blot analysis revealed a significant reduction in the HES1 protein levels in both HCT15 and SW480 cells following the combined treatment compared with treatment with 5-FU alone (1.59-fold reduction in HCT15, *P *= 0.0077; 1.28-fold reduction in SW480, *P *= 0.0423; Fig. 5C). Regarding JAG1 protein levels, HCT15 showed a greater reduction (1.88-fold, *P *= 0.0460), while SW480 exhibited a milder yet significant decrease (1.4-fold, *P *= 0.0346; Fig. 5D).

These findings suggest that the addition of DAPT to 5-FU treatment may effectively counteract the upregulation of key Notch-related components induced by 5-FU.

### Modulation of apoptosis- and stemness-related markers by 5-FU and DAPT in CRC cell lines

To further explore the molecular mechanisms associated with the cytotoxic effects of the combined treatment, we analysed key proteins related to cell-death pathways, namely PARP1 and LC3β.

**Figure 6:**
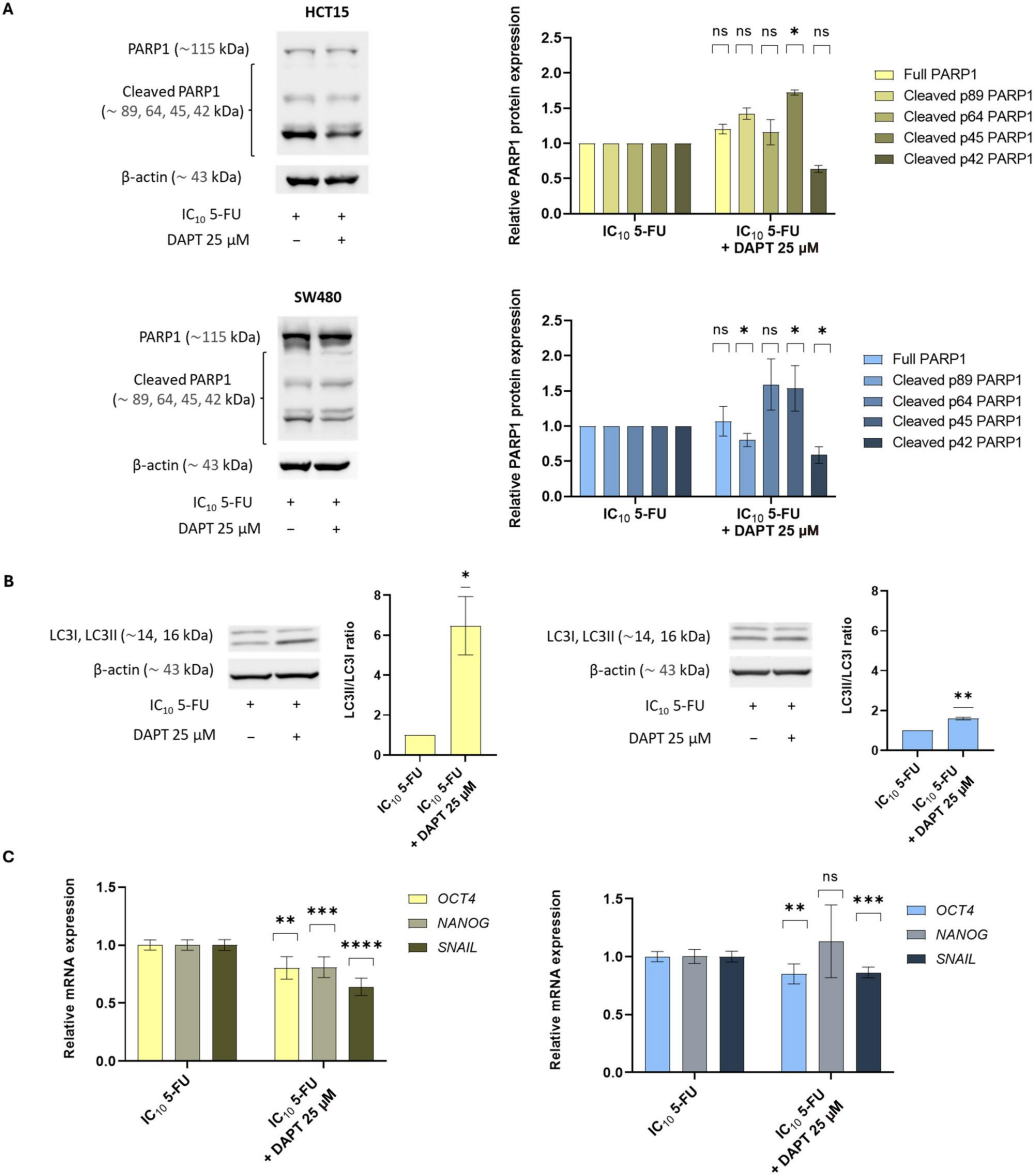
Effects of 5-FU alone or in combination with DAPT on cell-death-related proteins and stemness markers in CRC cell lines. Western blot analysis of cleaved (A) PARP1 and (B) LC3β in HCT15 and SW480 cells treated with 5-FU alone or in combination with DAPT for 72 h using the simultaneous-treatment schedule. In (A), HCT15 and SW480 cells are shown in the top and bottom images, respectively; in (B), HCT15 and SW480 are displayed on the left and right, respectively. β-actin was used as a loading control. Data were analysed by using a one-sample *t*-test. Results are presented as mean ± SD of at least two independent experiments. (C) Relative mRNA-expression levels of stemness-related genes in HCT15 (left) and SW480 (right) cells under the same treatment conditions. Gene expression was assessed by using RT–qPCR and normalized to the endogenous control *HPRT1*. Data were analysed by using a two-tailed unpaired Student’s *t*-test and are presented as mean ± SD of at least two independent experiments. Statistical significance is indicated by *P < 0.05, **P < 0.01, ***P < 0.001, ****P < 0.0001. ns, not significant; SD, standard deviation; 5-FU, 5-fluorouracil; CRC, colorectal cancer; DAPT, *N*-[*N*-(3,5-difluorophenacetyl-l-alanyl)]-*S*-phenylglycine *t*-butyl ester; IC_10_, inhibitory concentration reducing viability by 10%; HPRT1, Hypoxanthine phosphoribosyltransferase 1; JAG1, Jagged1; *OCT-4*, Octamer-binding protein 4; *NANOG*, Nanog Homeobox; *SNAIL*, Snail family transcriptional repressor 1; PARP1, poly(ADP-Ribose) polymerase 1; LC3B, microtubule-associated protein 1 light chain 3 beta.

Western blot analysis of PARP1 revealed multiple cleaved fragments that were indicative of apoptosis induction under both 5-FU alone and the combined treatment (Fig. 6A). Notably, the cleaved p45 PARP1 fragment was significantly increased in cells treated with the combination compared with those treated with 5-FU alone, with a 1.72-fold increase in HCT15 (*P *= 0.0216) and a 1.54-fold increase in SW480 (*P *= 0.0452). Interestingly, the levels of the smaller cleaved fragment (p42) were reduced following the combined treatment in both cell lines, with a statistically significant decrease observed in SW480 (1.72-fold reduction, *P *= 0.0397) and a non-significant trend in HCT15 (1.58-fold reduction, *P *= 0.0621).

Regarding autophagy, LC3β protein analysis revealed a clear increase in the LC3II/LC3I ratio after the combined treatment, indicating enhanced autophagic activity (Fig. 6B). The increase was especially notable in HCT15 cells, with a 6.47-fold elevation (*P *= 0.0230), while a more modest but still significant 1.60-fold increase was observed in SW480 (*P *= 0.0036). As LC3II formation results from the lipidation of LC3I, this shift suggests increased autophagosome formation.

Together, these findings indicate that the combination of 5-FU and DAPT triggers both apoptosis and autophagy in CRC cells, potentially contributing to its enhanced cytotoxic effect.

Finally, given the role of the Notch signalling pathway in the maintenance of cancer stem cells (CSCs), we examined whether the addition of DAPT to 5-FU could influence the expression of stemness- and epithelial–mesenchymal transition (EMT)-related genes. RT–qPCR analysis showed that the combination treatment significantly reduced the expression of *OCT-4*, *NANOG*, and *SNAIL* in HCT15 cells compared with those treated with 5-FU alone (1.25-fold, *P *= 0.0011; 1.23-fold, *P *= 0.0009; and 1.56-fold, *P *< 0.0001, respectively; Fig. 6C). In SW480 cells, no significant change was observed for *NANOG*; however, *OCT-4* and *SNAIL* were modestly but significantly downregulated (1.17-fold, *P *= 0.0034; and 1.16-fold, *P *= 0.0005, respectively). However, these changes were relatively minor for most of the genes studied, so further studies will be needed to determine the impact of the treatment combination on CSC features.

### Secretome derived from 5-FU- and DAPT-treated CRC cells impairs angiogenesis *in vitro*

To assess the impact of CRC-cell secretomes on angiogenic potential, we performed tube-formation assays by using HUVECs cultured with CM derived from HCT15 and SW480 cells previously treated for 24 h with 5-FU (IC_10_), DAPT (25 µM), or their combination. Importantly, the 5-FU dose used corresponds to the IC_10_ at 72 h; however, for the preparation of CM, CRC cells were exposed to treatment for only 24 h, during which no cytotoxic effects were observed under our experimental conditions.

**Figure 7:**
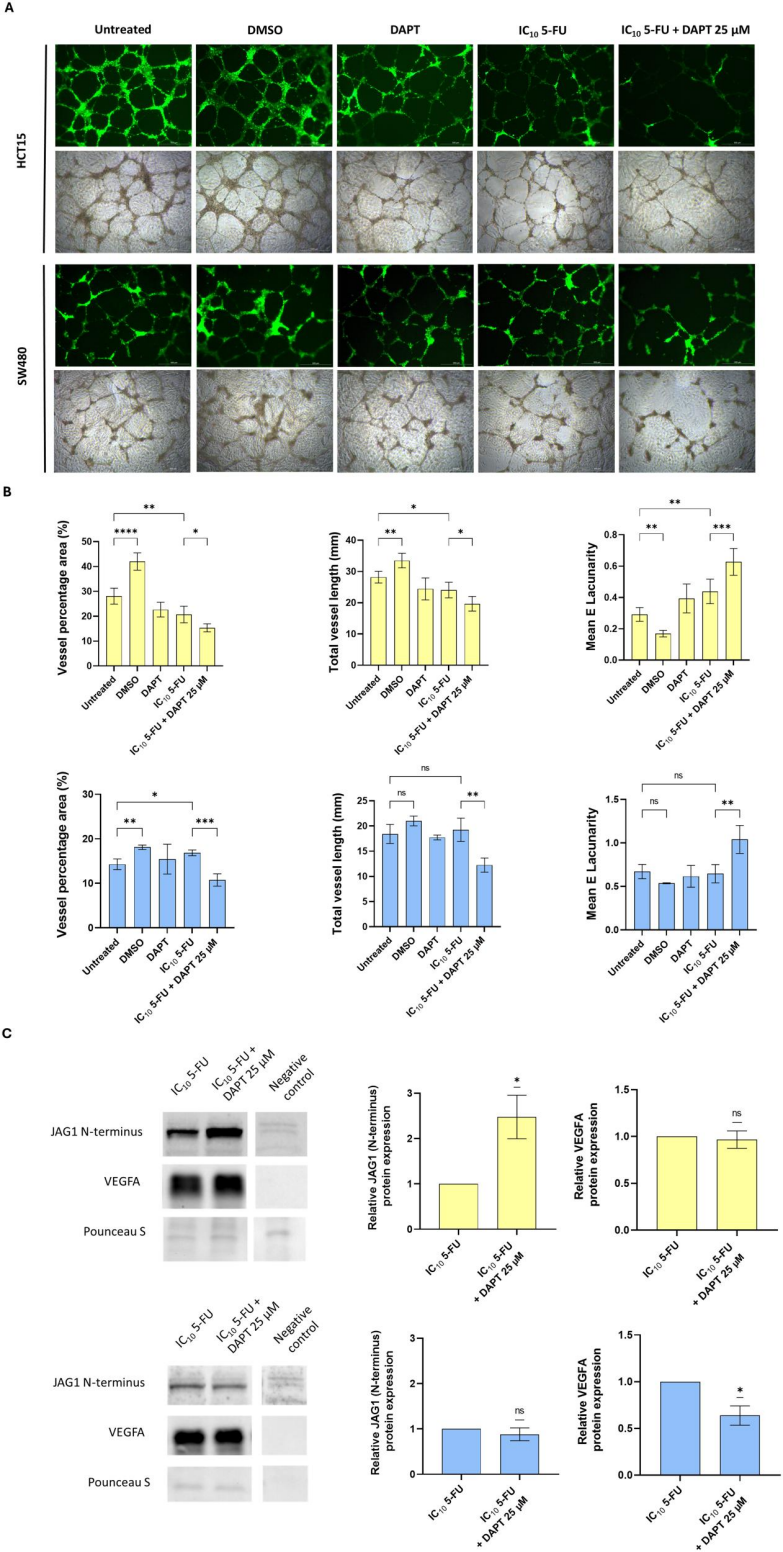
Attenuation of tube-forming capacity in HUVECs following incubation with CM from treated CRC cells. (A) Representative optical and fluorescent microscopy images of tube formation by HUVECs after 12 h of incubation with CM collected from HCT15 and SW480 cells treated with the indicated agents for 24 h. Images were acquired by using a 4× objective. (B) Quantification of tube formation based on the vessel percentage area, total vessel length, and mean lacunarity of the HUVECs incubated as indicated in (A). HCT15 and SW480 results are shown in the top and bottom panels, respectively. Data were analysed by using a one-way ANOVA test. Results are presented as mean ± SD of two independent experiments. (C) Western blot analysis of soluble JAG1 and VEGFA in CM collected from HCT15 (top) and SW480 (bottom) cells treated with 5-FU alone or in combination with DAPT for 24 h at subtoxic concentrations. Medium not exposed to cells was used as a negative control. β-actin was used as a loading control. Differences between conditions were analysed by using a one-sample *t*-test. Results are presented as mean ± SD of three independent experiments. Statistical significance is indicated by *P < 0.05, **P < 0.01, ***P < 0.001, ****P < 0.0001. ns, not significant; CM, conditioned media; SD, standard deviation; 5-FU, 5-fluorouracil; CRC, colorectal cancer; DAPT, *N*-[*N*-(3,5-difluorophenacetyl-l-alanyl)]-*S*-phenylglycine *t*-butyl ester; DMSO, dimethyl sulfoxide; IC_10_, inhibitory concentration reducing viability by 10%; HUVEC, human umbilical vein endothelial cell; JAG1, Jagged1; VEGFA, vascular endothelial growth factor A.

In HCT15-derived CM, the combination of 5-FU and DAPT significantly impaired angiogenic features compared with CM from HCT15 treated with 5-FU alone. Specifically, a significant reduction was observed in both the vessel percentage area (*P *= 0.0455) and the total vessel length (*P *= 0.029), along with a notable increase in the mean lacunarity (*P *= 0.0004), indicating reduced vessel complexity (Fig. 7A and B). Similar results were obtained with SW480-derived CM, showing a significant decrease in the vessel area (*P *= 0.0002) and vessel length (*P *= 0.0031) and an increase in the lacunarity (*P *= 0.0051).

Interestingly, CM from DMSO-treated cells induced a pro-angiogenic effect compared with untreated controls—an effect not observed in CM from DAPT-treated cells. In HUVECs incubated with CM from DMSO-treated HCT15 cells, the vessel area and vessel length were significantly increased (*P *< 0.0001 and *P *= 0.0023, respectively), while the lacunarity was significantly reduced (*P *= 0.0070). In contrast, CM from DMSO-treated SW480 cells did not significantly alter the vessel length or lacunarity (*P *= 0.2755 and *P *= 0.3954, respectively), although the vessel area was slightly increased (*P *= 0.0047). This unexpected result suggests that short-term exposure to DMSO, while generally considered inert at low concentrations, may modulate the tumour-cell secretome in a manner that enhances angiogenic signalling.

Given that Notch signalling is canonically transmitted through cell–cell contact, we aimed to understand the molecular mechanism underlying the differences observed between 5-FU alone and the combination with DAPT. To explore potential mediators of this angiogenic modulation, we analysed the presence of sJAG1 and VEGFA in the CM. Soluble JAG1, corresponding to the N-terminal domain of the protein, was detected in both HCT15 and SW480 CMs but not in the negative control, confirming secretion by tumour cells (Fig. 7C). Interestingly, HCT15 cells treated with the 5-FU and DAPT combination exhibited a significant increase in sJAG1 levels compared with those treated with 5-FU alone (2.48-fold, *P *= 0.0336). However, no significant differences in sJAG1 levels were observed between the treatments in SW480 cells.

We next analysed the levels of VEGFA—a major pro-angiogenic cytokine—in the same CM as an alternative angiogenic mediator. In SW480-derived CM, the combination treatment led to a significant reduction in the VEGFA protein levels (1.57-fold reduction, *P *= 0.0258) compared with treatment with 5-FU alone, while the VEGFA levels remained unchanged across the treatment conditions in HCT15.

These findings suggest that the anti-angiogenic effects observed following combined 5-FU and DAPT treatment are mediated through distinct mechanisms, depending on the CRC cell line. In HCT15 cells, increased levels of sJAG1 may contribute to impaired angiogenesis, while, in SW480 cells, reduced VEGFA secretion appears to play a more prominent role. Nonetheless, additional experimental evidence will be required to directly assess the impact of sJAG1 on the tube-forming capacity of HUVECs.

## Discussion

**Figure 8:**
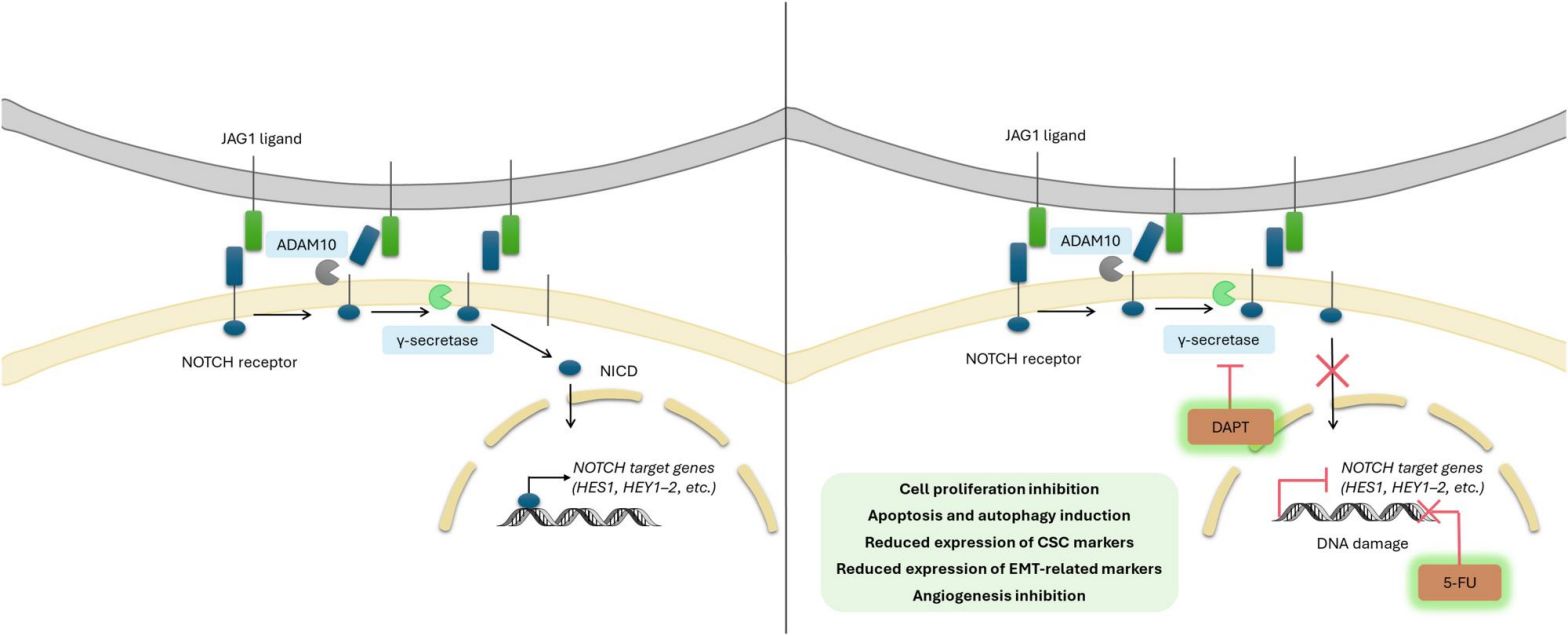
Schematic illustration of Notch signalling in untreated (left) and 5-FU plus DAPT-treated (right) CRC cells. In untreated cells (left), binding of the JAG1 ligand to a Notch receptor triggers ADAM10-mediated cleavage followed by γ-secretase processing, generating the NICD, which translocates to the nucleus and activates Notch target genes (e.g. *HES1*). In cells exposed to the combination of 5-FU and DAPT (right), γ-secretase inhibition prevents NICD release and reduces the expression of Notch-related genes. This dual treatment enhances the effects initiated by 5-FU, such as DNA damage and reduced proliferation, while Notch inhibition further increases apoptosis and autophagy, decreases CSC and EMT marker expression, and reduces pro-angiogenic signalling, collectively potentiating the antitumour activity of 5-FU. CRC, colorectal cancer; 5-FU, 5-fluorouracil; DAPT, *N*-[*N*-(3,5-difluorophenacetyl-l-alanyl)]-*S*-phenylglycine *t*-butyl ester; JAG1, Jagged1; ADAM10, a disintegrin and metalloproteinase domain-containing protein 10; NICD, Notch intracellular domain; HES1, Hes family bHLH transcription factor 1; HEY1–2, Hes-related family bHLH transcription factor with YRPW motif 1 and 2; CSC, cancer stem cell; EMT, epithelial–mesenchymal transition.

5-Fluorouracil-based regimens remain a cornerstone of metastatic CRC therapy, yet therapeutic resistance continues to limit the long-term benefits. Anti-angiogenic agents such as bevacizumab improve outcomes in selected patients, but reliable biomarkers capable of predicting benefit remain scarce [3, 28, 29]. Increasing evidence implicates JAG1-mediated Notch signalling in tumour progression, angiogenesis, and therapy adaptation [19, 30, 31], yet its relevance in the context of bevacizumab-containing regimens and the extent to which 5-FU itself modulates this pathway remain insufficiently understood. In this study, we show that the addition of bevacizumab to the therapeutic regimen significantly improves PFS and TTP, and that tumour JAG1 expression stratifies the clinical outcome specifically in these patients. We also identify a 5-FU-induced activation of the JAG1/Notch axis in CRC cells and demonstrate that γ-secretase inhibition dampened this activation, thereby enhancing the cytotoxic and anti-angiogenic effects of 5-FU (Fig. 8). Together, these results bridge clinical observations with mechanistic *in vitro* findings and highlight the biological relevance of the JAG1/Notch axis in the treatment response.

In our clinical cohort, patients with metastatic CRC treated with bevacizumab-containing regimens showed clear survival benefits, consistently with robust evidence from clinical trials demonstrating the efficacy of this anti-angiogenic agent when combined with chemotherapy [9, 32, 33]. This concordance serves as an internal validation step, ensuring that our cohort behaves as expected clinically and is therefore suitable for subsequent molecular correlation analyses. Within this context, we aimed to examine the potential prognostic value of tumour JAG1 expression. Although JAG1 levels were not associated with outcomes in the full cohort or in patients receiving chemotherapy alone, high JAG1 expression identified a subgroup of bevacizumab-treated patients with shorter PFS and TTP. The effect sizes were large and the survival curves were clearly separated, indicating a clinically meaningful difference despite the lack of statistical significance, most likely due to the small size of the bevacizumab-treated subgroup. This suggests that the association is likely real but underpowered, supporting the idea that JAG1 may function as a biomarker of bevacizumab benefit rather than a general prognostic biomarker in CRC. JAG1 has been implicated in adaptive angiogenesis and resistance to anti-VEGF therapies [34, 35], raising the possibility that elevated tumour JAG1 promotes alternative signalling routes that remain active despite VEGF blockade. These observations support JAG1 as a candidate for prospective biomarker validation in larger cohorts. Although tumour sidedness is an established prognostic factor in CRC, preliminary Cox regression including the tumour location showed no associations with survival in our dataset (data not shown). In the absence of correlation and given the limited sample size, further stratification would have substantially reduced the statistical power; accordingly, our analyses did not distinguish between right- and left-sided tumours.

Several attempts to translate Notch-targeting strategies into clinical benefit by using GSIs have produced discordant results in CRC [22, 36, 37], underscoring the complexity of pathway inhibition in this context. To mechanistically explore how JAG1/Notch activity may influence treatment response, we investigated the effects of 5-FU on Notch pathway activation and evaluated whether pharmacological Notch inhibition modifies the cytotoxic and angiogenic consequences of 5-FU exposure. We observed that 5-FU upregulated the canonical Notch target *HES1*, which is a well-established transcriptional readout of pathway activation due to its rapid response to NICD release [30]. The upregulation of *JAG1* was more modest and cell-line-dependent, with a clearer induction in HCT15 cells. This pattern is consistent with the context-specific regulation of Notch ligands and with reports showing chemotherapy-driven JAG1 expression [38–40]. These findings support the idea that Notch activation forms part of a stress-adaptive response to 5-FU and may contribute to chemoresistance. Importantly, DAPT effectively attenuated this 5-FU-induced activation at both transcript and protein levels, in line with prior studies showing that Notch inhibition can enhance chemosensitivity.

This molecular inhibition translated into functional synergy. Combined 5-FU and DAPT treatment resulted in a significant synergistic reduction in cell viability in both CRC models, consistently with reports that GSIs potentiate the effects of fluoropyrimidines [41–44]. The combination triggered apoptosis, evidenced by accumulation of the 45-kDa PARP1 fragment. Notably, this accumulation suggests the involvement of proteases other than caspase-2, such as cathepsins or calpains [45]. Moreover, the combinatorial treatment activated autophagy, reflected by an increased LC3II/LC3I ratio. Previous studies support these observations, showing that DAPT induces apoptosis and cell-cycle arrest in CRC cells [46, 47] and enhances 5-FU-driven PARP1 cleavage in the CRC cell line HT29 [44]. While the role of DAPT in autophagy is less well defined in CRC, Notch inhibition has been associated with increased LC3II and Beclin1 levels in other tumour models [48] and apoptosis and autophagy frequently occur sequentially and are mechanistically interconnected [49]. Our results therefore indicate that Notch contributes to CRC-cell survival under 5-FU stress, and that the dual activation of apoptosis and autophagy may underlie the enhanced cytotoxicity observed with the combination.

Given the role of Notch in maintaining stemness and EMT plasticity [50], we evaluated associated transcriptional markers. The combination of 5-FU and DAPT led to modest but significant reductions in *OCT-4*, *NANOG*, and *SNAIL* in HCT15 cells, with weaker effects in SW480 cells. Although these small changes should be interpreted with caution, they align with reports linking Notch inhibition to reduced stem-like features and EMT-associated plasticity in CRC models [50–53]. Discrepancies with studies showing stronger suppression may reflect variations in the GSI potency, drug scheduling, or intrinsic heterogeneity across CRC cell lines. Further work using genetic inhibition and alternative GSIs will be needed to clarify whether this combination has a meaningful impact on CSC behaviour.

A novel aspect of this study is the impact of 5-FU and DAPT on the CRC secretome and its angiogenic influence. CM from combination-treated cell significantly impaired HUVEC-tube formation, reducing the vessel area and total vessel length, and increasing the lacunarity. Beyond the classical juxtacrine model of canonical Notch signalling, accumulating evidence indicates that soluble forms of Notch ligands, including sJAG1, can modulate pathway activity [54–56]. In our study, HCT15 cells secreted increased levels of sJAG1—a form known to act as a dominant-negative regulator by disrupting ligand–receptor interactions. This mechanism provides a plausible explanation for the impaired angiogenic response observed in HUVECs exposed to sJAG1-enriched CM. In contrast, SW480 cells showed no change in sJAG1, but exhibited a marked reduction in VEGF secretion following the combination treatment, suggesting that VEGF-dependent rather than sJAG1-mediated signalling underlies the angiogenic impairment in this cell line. These divergent mechanisms illustrate the heterogeneity of CRC secretome responses and highlight an underexplored layer of Notch-mediated intercellular communication. Functional studies, such as sJAG1 blockade or knockdown, will be required to confirm whether sJAG1 directly contributes to the anti-angiogenic phenotype.

This study has several limitations. The sample size of the bevacizumab-treated subgroup restricts the statistical power to detect survival differences with high confidence; larger multicentre datasets will be needed to validate JAG1 as a prognostic biomarker. The mechanistic assays relied on pharmacological inhibition, and the genetic perturbation of specific Notch receptors and ligands will be necessary to delineate their individual contributions. Finally, the lack of *in vivo* validation limits our ability to determine whether the synergistic efficacy of 5-FU and DAPT translates into improved tumour control in a physiological context. These limitations underscore important directions for future research.

In summary, our work reveals a dual clinical and mechanistic role for JAG1/Notch signalling in CRC. We show that high tumour JAG1 expression is associated with poorer outcomes in bevacizumab-treated patients, that 5-FU activates Notch signalling in CRC cells, and that γ-secretase inhibition partially reduces this activation, synergistically enhancing both the cytotoxic and anti-angiogenic effects of 5-FU. The finding that CRC cells secrete sJAG1 and that this secretion is modulated by treatment adds a previously overlooked facet to Notch-dependent intercellular communication. Collectively, these results support further exploration of Notch pathway inhibitors as adjuncts to 5-FU-based therapies, with future studies required to validate these findings through genetic, functional, and *in vivo* approaches.

## Supplementary Material

goag012_Supplementary_Data
