# Supplementary material for: Tumour Jagged1 expression as a prognostic marker of bevacizumab response and modulation of 5-fluorouracil efficacy through γ-secretase inhibition in colorectal cancer
Source: Gastroenterol Rep (Oxf). 2026 Mar 10;14:goag012. doi: 10.1093/gastro/goag012 (PMC12975003; doi:10.1093/gastro/goag012)
Supplement: goag012_Supplementary_Data [file goag012_supplementary_data.zip › GR-2025-472.R1_Supplementary_data.docx]

**Supplementary data**

**Supplementary Figure 1**. Stability value and ranking of candidate reference genes by the different RefFinder algorithms. To determine the most stable reference gene, four housekeeping genes (*HPRT1, GAPDH, B2M,* and *MTR*) were evaluated in (A) HCT15 and (B) SW480 cells using RefFinder, which combines four distinct algorithms (Delta Ct method, geNorm, NormFinder, and BestKeeper) to provide a comprehensive stability ranking. Based on this analysis, *HPRT1* was identified as the most stable reference gene across the experimental conditions tested (Untreated, IC_10_ 5-FU, and IC_10_ 5-FU + DAPT). 5-FU, 5-fluorouracil; B2M, beta–2–microglobulin; GAPDH, glyceraldehyde-3-phosphate dehydrogenase; HPRT1*,* hypoxanthine phosphoribosyltransferase 1; MTR, 5-methyltetrahydrofolate–homocysteine methyltransferase; IC_10_, inhibitory concentration reducing viability by 10%; ΔCt, Delta Ct method.

**Supplementary Table 1.** Primers used for the housekeeping genes included in the RefFinder analysis.

| **Gene** | **Primer sequence** | **Tm (°C)** |
| --- | --- | --- |
| *HPRT1* | F- TGACACTGGCAAAACAATGCA | 67.5 |
|  | R- GGTCCTTTTCACCAGCAAGCT | 66.6 |
| *MTR* | F- CACATTTGATGAGCTTGTTG | 59.3 |
|  | R- ATGAGTAAGATATCAACCCCG | 59.4 |
| *GAPDH* | F- CTCTGATTTGGTCGTATTGG | 59.6 |
|  | R- GTAAACCATGTAGTTGAGGTCC | 55.1 |
| *B2M* | F- AAGGACTGGTCTTTCTATCTC | 55.6 |
|  | R- GATCCCACTTAACTATCTTGG | 56.7 |

B2M, beta–2–microglobulin; GAPDH, glyceraldehyde-3-phosphate dehydrogenase; HPRT1*,* hypoxanthine phosphoribosyltransferase 1; MTR, 5-methyltetrahydrofolate–homocysteine methyltransferase; Tm, melting temperature.
