# Supplementary figures and images for: Tumour Jagged1 expression as a prognostic marker of bevacizumab response and modulation of 5-fluorouracil efficacy through γ-secretase inhibition in colorectal cancer
Source: Gastroenterol Rep (Oxf). 2026 Mar 10;14:goag012. doi: 10.1093/gastro/goag012 (PMC12975003; doi:10.1093/gastro/goag012)

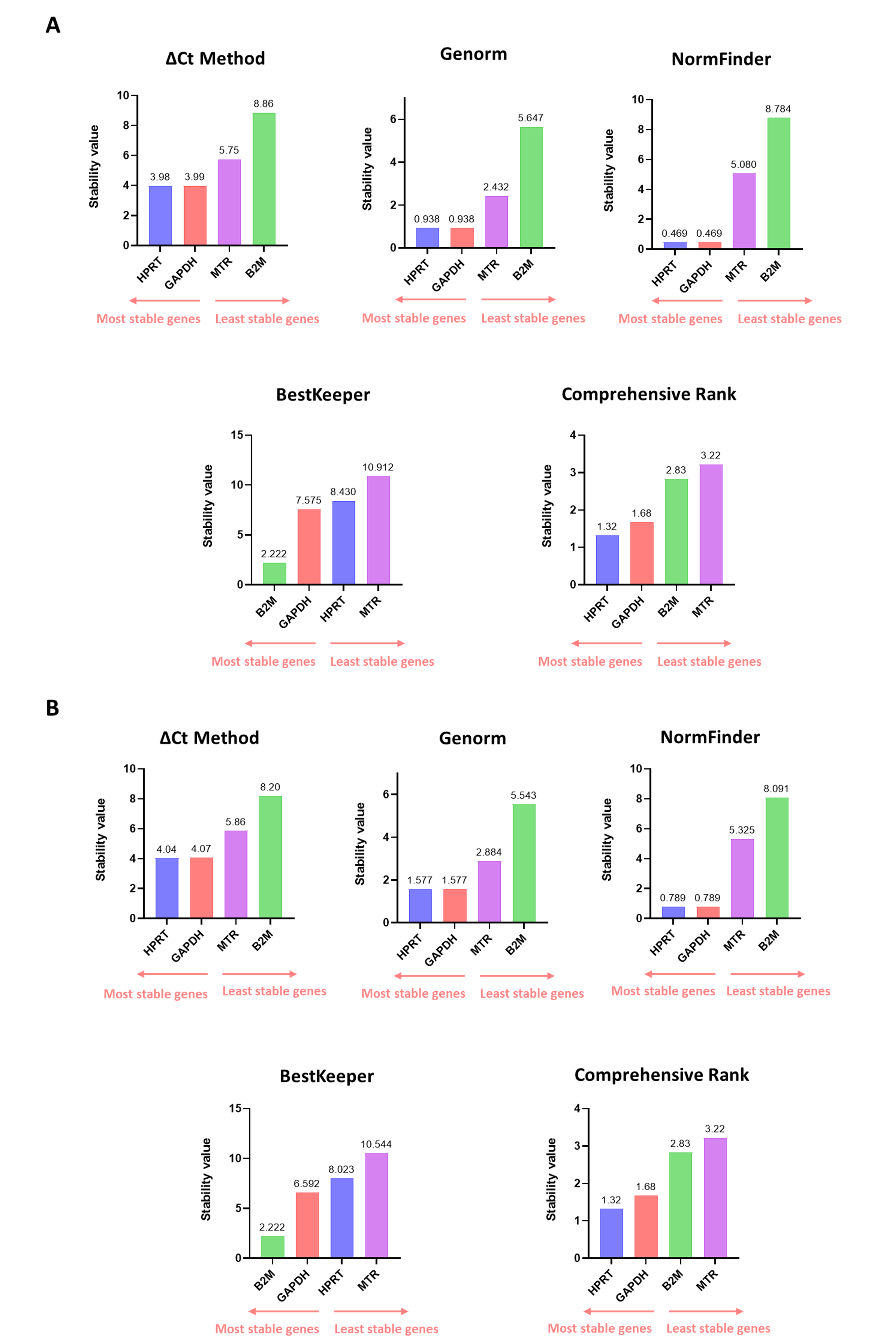

Supplement: goag012_Supplementary_Data [file goag012_supplementary_data.zip › Supplementary_Figure_1.tif]
